# Ancestral Polymorphisms and Sex-Biased Migration Shaped the Demographic History of Brown Bears and Polar Bears

**DOI:** 10.1371/journal.pone.0078813

**Published:** 2013-11-13

**Authors:** Shigeki Nakagome, Shuhei Mano, Masami Hasegawa

**Affiliations:** 1 Risk Analysis Research Center, The Institute of Statistical Mathematics, Tachikawa, Tokyo, Japan; 2 Department of Mathematical Analysis and Statistical Inference, The Institute of Statistical Mathematics, Tachikawa, Tokyo, Japan; 3 School of Life Sciences, Fudan University, Shanghai, China; University of Utah, United States of America

## Abstract

Recent studies have reported discordant gene trees in the evolution of brown bears and polar bears. Genealogical histories are different among independent nuclear loci and between biparentally inherited autosomal DNA (aDNA) and matrilineal mitochondrial DNA (mtDNA). Based on multi-locus genomic sequences from aDNA and mtDNA, we inferred the population demography of brown and polar bears and found that brown bears have 6 times (aDNA) or more than 14 times (mtDNA) larger population sizes than polar bears and that polar bear lineage is derived from within brown bear diversity. In brown bears, the effective population size ratio of mtDNA to aDNA was at least 0.62, which deviated from the expected value of 0.25, suggesting matriarchal population due to female philopatry and male-biased migration. These results emphasize that ancestral polymorphisms and sex-biased migration may have contributed to conflicting branching patterns in brown and polar bears across aDNA genes and mtDNA.

## Introduction

Genealogical discordance is highlighted in phylogenetic relationships between brown bears (*Ursus arctos*) and polar bears (*U*. *maritimus*). Phylogenetic analyses based on mitochondrial DNA (mtDNA) indicate that the root of polar bear lineage is within brown bear diversity, representing a paraphyletic relationship [1], [2]. In contrast, two recent studies based on autosomal DNA (aDNA) found early divergence of polar bears from the common ancestor (*i*.*e*. monophyly) of brown bears by exploring one species tree from multi-locus data [3], [4]. Hailer et al. [3] sequenced 14 independent aDNA loci in geographically diverse samples from brown bears and polar bears and estimated that polar bears split off 603 thousand years ago (ka), followed by brown bear divergence at 125 ka. The authors concluded that discrepancies in phylogenies between aDNA and mtDNA were the result of mtDNA introgression from brown bears to polar bears due to hybridization. Based on genome scale sequence data, Miller et al. [4] suggested the ancient divergence of polar bears from brown bears with an estimated split time of 4 to 5 million years ago (MYA). The authors concluded that extensive gene flow had maintained the genetic relationship between brown bears and polar bears until 200 to 100 ka and that polar bear mtDNA was replaced with brown bear mtDNA.

**Figure 1:**
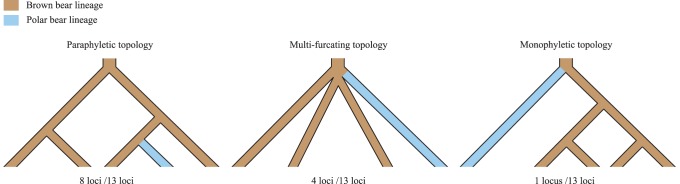
Schematic topologies of aDNA gene trees from 13 loci. Based on the phylogenetic relationship between brown bears and polar bears (see Figure S1), gene trees are classified into paraphyletic, multi-furcating, and monophyletic topologies. The paraphyletic topology includes eight out of 13 loci (*ABCA1*, *SEL1L3*, *PREX2*, *SPTBN1*, *OSTA*, *CCDC90B*, *SPTA1*, and *IGSF22*). Only one locus, *AZIN1*, supports the monophyletic topology. The remaining four loci (*GGA3*, *ATP12A*, *TRAPPC10*, and *SCN5A*) are assigned into the multi-furcating topology.

In contrast to previous reports on discordant gene trees between aDNA and mtDNA, Nakagome et al. [5] pointed out that genealogical histories from aDNA genes were compatible with mtDNA history and that the majority of discrepancies could be traced to stochastic processes in which ancestral polymorphisms and subsequent incomplete lineage sorting shaped a unique gene genealogy. Based on sequence data from Hailer et al. [3], Nakagome et al. [5] simulated the stochastic process under the coalescent model [6] and tested whether genealogy at each locus significantly deviated from the paraphyletic model of mtDNA phylogeny. The results were completely different from Hailer et al. [3], and none of the aDNA genes could reject the paraphyletic model. Indeed, we observed conflicting gene genealogies among the aDNA loci (see Figure 1 and Figure S1), which likely result from different patterns of ancestral polymorphisms and incomplete lineage sorting. Gene trees from eight and four out of 13 loci analyzed in the previous study [5] showed paraphyletic and multi-furcating topology, respectively. Although one locus, *AZIN1*, depicted monophyletic topology, only one mutation defined the brown bear clade. The origin of brown bears and polar bears remains unresolved, and further studies are required to reconcile these dramatically different results.

There are two types of discrepancies in the evolution of brown bears and polar bears, one among unlinked aDNA genes and the other between aDNA and mtDNA. The first discrepancy can be attributed to ancestral polymorphisms and incomplete lineage sorting [7]. Given enough times (∼5*N*_e_ generations, where *N*_e_ is the effective number of chromosomes), DNA sequences in a population are likely to coalesce within each population and any loci can be expected to depict a consistent gene tree with the species tree. Because *N*_e_ determines the amount of ancestral polymorphisms and the probability of coalescence in a population, gene tree discordance among loci depends on *N*_e_. In contrast, the second discrepancy may correlate with behavioral traits of female philopatry and male-biased dispersal in brown bears [8], [9]. A recent study pointed out that sex-biased migration is a plausible cause of inconsistent bear phylogenies among aDNA, mtDNA, and sex-linked DNA [10]. To address these issues, we inferred a demographic history of brown and polar bears using aDNA and mtDNA.

## Materials and Methods

### DNA Sequence Data

We used two different datasets of population-based sequences from autosomal DNA (aDNA) genes and complete mitochondrial DNA (mtDNA) (Table S1). All sequence data were downloaded from the European Molecular Biology Laboratory (EMBL) Nucleotide Sequence Database (http://www.ebi.ac.uk/embl/) and GenBank (http://www.ncbi.nlm.nih.gov/genbank/). aDNA sequences included brown bears (2*n* = 36) and polar bears (2*n* = 36) from 14 loci [3]. Brown bear sequences were derived from broad geographic regions in Norway, Romania, Sweden, Bulgaria, U.S.A., and Canada (see Table S1 in Hailer et al. [3]). mtDNA sequences were from brown bears (*n* = 9) and polar bears (*n* = 26). To infer an ancestral allele at each segregating site among brown and polar bears, we used orthologous sequences from the giant panda for 14 aDNA loci [11] and the complete mtDNA sequences of one giant panda (the accession number: EF212882) and two American black bears (JX196366; NC003426).

In the mtDNA genome (except for the D-loop region), some of the sequences contained multiple substitutions, which did not follow the infinite site model in population genetic analyses. Under the infinite site model, one mutation event can occur at a previously un-mutated site and can be disrupted by back mutation or recurrent mutation events. We detected incompatibilities between mutated sites using the four-gamete test implemented in DnaSP version 5.1 [12], [13]. If mutations are incompatible, we can infer recurrent mutation at the sites because mtDNA is the haploid, non-recombination sequence. To minimize the effects of multiple substitutions on population genetic analyses, we generated two mtDNA datasets by excluding (1) sequences (Set-I) or (2) multiple substitution sites (Set-II). (1) We excluded sequences with multiple substitutions from brown bears or polar bears and aligned the mtDNA sequences. However, the aligned sequences still contained multiple substitutions spanning from the 8,269 nucleotide position (np) to 15,439 np, and approximately half of the mtDNA sequences were trimmed. As a result, the 8,268 bp mtDNA dataset (the sequence from 1 np to 8,268 np) was comprised of data from four brown bears and ten polar bears (Table S1). (2) We excluded multiple substitution sites from alignment of 35 mtDNA sequences (nine from brown bears and 26 from polar bears), with a total sequence length of 15,403 bp.

### Reconstruction of Gene Trees for aDNA Loci

We estimated gene genealogy for 13 aDNA loci from brown bears (2*n* = 36) and polar bears (2*n* = 36) using GENETREE [14]. Because the *LRGUK* locus included a high frequency of recombinant sequences and did not follow the infinite site model, this locus was excluded from GENETREE analysis. Genealogy was deduced from the path of mutations to the most recent common ancestor (MRCA) under the infinitely many-site model [15]. Under this model, a gene is thought to be an infinite sequence of completely linked sites where mutations occur at sites that have never experienced mutations before. Given the gene tree, we computed maximum likelihood estimates for the population mutation rate and generated the empirical distribution of time to MRCA (*T*_MRCA_), as described in a previous study [5].

### Bayesian Inference for Demographic History

To estimate demographic parameters, we applied kernel approximate Bayesian computation (kernel-ABC) [16], [17] to the sequence data from aDNA or mtDNA. ABC is a statistical framework to obtain approximation of a posterior estimate without likelihood function. Sequence data are summarized into a set of summary statistics. The advantage of kernel-ABC is that the cost of computing time is independent of the dimension of data, and thus high-dimensional summary statistics can be incorporated into the analyses. In this study, we used site frequency spectrum (SFS) and haplotype frequency spectrum (HFS) as the set of summary statistics. SFS consists of the site frequency, *ξ_i_*, which is the number of segregating sites in which the mutant nucleotide is present on *i* sequences in the sample. SFS reflects a pattern of mutations among segregating sites and refines a partition of data determined by conventional summary statistics, such as the number of segregating sites, nucleotide diversity [18], and Tajima’s D [19]. A previous study showed that SFS can improve approximation of the posterior estimate given the full data compared with conventional statistics [16]. However, HFS consists of the haplotype frequency, *δ_j_*, which is the number of haplotypes observed in *j* sequences in the samples. HFS can account for recombination patterns at a locus. Because sequence data from aDNA include many recombinants, the combination of SFS and HFS provides more detailed information than SFS alone. We summarized the sequence data into SFS for brown bears (SFS_uar_) or polar bears (SFS_uma_) and two-dimensional HFS (2D-HFS) in which each haplotype was shared between brown bears and polar bears or specific to the population. For aDNA data, we calculated SFS_uar_, SFS_uma_, and 2D-HFS for each locus (Figures S2a–S2n) and merged the data into a set of summary statistics across 14 loci. The allelic state (ancestral/derived allele) at each segregating site was determined by alignment with an orthologous giant panda sequence. As mtDNA is a haploid genome, we generated 2D-SFS in which each derived allele was shared between or specific to either brown bears or polar bears (Figures S2o and S2p). Because it is generally difficult to determine the allelic state using the giant panda sequence due to a higher mutation rate in mtDNA than aDNA, we used two mtDNA sequences from American black bears with phylogenetic positions that are closer to brown and polar bears compared with the giant panda [20]. If both American black bear sequences had the same allele for the segregating site as brown and polar bears, it was defined as the ancestral allele. Otherwise, we used the allele that was consistent with the giant panda sequence. Although some of the sites may contain back or recurrent mutations, most of the sites likely follow the infinite site model. Based on the observed summary statistics for aDNA and mtDNA, we estimated posterior means of parameters by kernel-ABC.

The demographic model represents population divergence between brown bears and polar bears, and the parameters in the model are the effective population size in brown bears (*N*_uar_), polar bears (*N*_uma_), and divergence time between brown bear and polar bear populations (*T*). Prior distributions for each parameter were represented as a log-normal distribution (*LN*) with a variance as a square of the mean (*μ*). We used different sets of prior distributions between aDNA and mtDNA as follows: *N*_uar_∼*LN*(*μ* = 30000, *μ* ^2^ = 30000^2^), *N*_uma_∼*LN*(10000, 10000^2^), and *T*∼*LN*(100000, 100000^2^) for aDNA and *N*_uar_∼*LN*(200000, 200000^2^), *N*_uma_∼*LN*(10000, 10000^2^), and *T*∼*LN*(30000, 30000^2^) for mtDNA. The kernel-ABC algorithm is as follows [16], [17]:

**for** *i* = 1 to 20,000 **do**

1. Sample a parameter set of ***θ*** *_i_* = (*N*_uar*i*_, *N*_uma*i*_, *T_i_*) from prior distributions.
2. Simulate data using ***θ*** *_i_*.
3. Compute summary statistics (***s*** *_i_*) for the data.

**end for**

1. Compute posterior means of the parameters as a predictor by the kernel ridge regression of ***θ*** *_i_* onto ***s*** *_i_* based on {***θ*** *_i_*, ***s*** *_i_*}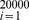.

We used the Gaussian radial base function kernel, 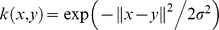. The bandwidth (*σ* ^2^) in the kernel function and regularization parameter (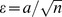, where *n* is the number of simulations) were selected based on previously described 10-fold cross validation [16], [17]. This algorithm was repeated 100 times and the mean and standard deviation (S.D.) of the posterior mean estimate for each parameter were calculated.

All simulations were performed using the program package *ms,* which generates samples from the coalescent model [21]. In coalescent simulation of aDNA genes, we assumed that a recombination rate was equal to a mutation rate. The mutation rate was calculated from the average number of substitutions between the giant panda and brown and/or polar bears assuming that divergence of the giant panda and brown/polar bears is 12 MYA, which represents the oldest remains from a member of the giant panda lineage and is compatible with the molecular clock estimate [22], [23]. For aDNA genes, we averaged mutation rates across 14 loci to derive an estimate of 1.314×10^−8^ bp/site/generation. The number of substitutions in mtDNA was corrected by the Tajima-Nei distance model [24], and the mutation rate was estimated to be 7.036×10^−8^ bp/site/generation.

We assessed dependence of posterior estimates on prior conditions using sequence data from aDNA. Ten *LN*(*μ*, *μ* ^2^) types were given for each parameter: *μ* = (5000, 20000, 35000, 50000, 65000, 80000, 95000, 110000, 125000, 140000) for *N*_uar_ or *N*_uma_ and *μ* = (25000, 50000, 75000, 100000, 125000, 150000, 175000, 200000, 225000, 250000) for *T*. The fixed conditions were defined as *N*_uar_∼*LN*(30000, 30000^2^), *N*_uma_∼*LN*(10000, 10000^2^), or *T*∼*LN*(100000, 100000^2^). Ten different priors were provided for one parameter, while other parameters were sampled from the fixed conditions. The number of simulations was *n* = 10,000 for a set of prior distributions, and the mean and S.D. for the posterior estimate were calculated from 100 replications with *n* = 10,000.

### Test of Natural Selection in mtDNA

To detect signals of natural selection in mtDNA sequences (Set I and Set II) in brown bears, we calculated Tajima’s D [19], Fu and Li’s D and F [25], and Fay and Wu’s H [26] using DnaSP version 5.1 [12]. American black bear sequences were used as outgroup to determine ancestral states of alleles at segregating sites. The significance of these statistics was evaluated by the null distribution of 10,000 coalescent simulations under the constant size model using DnaSP [12].

### Estimation of T_MRCA_

We calculated *T*_MRCA_ in the genealogy between each of the geographic lineages in brown bears (Kenai, Kodiak, France, or ABC Islands) and polar bear lineages by kernel-ABC. mtDNA sequence data for Set-I and Set-II were summarized into 2D-SFS (Figures S3 and S4). The demographic parameter is *T*_MRCA_ = *T_n_*+*T_n_*_-1_+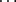
+ *T*_2_, where *T_i_* follows an exponential distribution with the parameter of *i*(*i* –1)/2 under the coalescent model. Based on the algorithm described in the previous section, we generated samples of {*T*_MRCA*i*_, ***s*** *_i_*}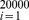 under the demographic model with estimated values of *N*_uar_, *N*_uma_, and *T* and estimated the posterior mean of the parameter. Similarly, we computed *T*_MRCA_ in the genealogy of polar bear lineages using SFS summary statistics from Set-I and Set-II. The demographic model had a constant population size of *N*_uma_.

## Results and Discussion

The demographic model assumed in this study is population divergence between brown bears and polar bears, including three parameters of effective population size in brown bears (*N*_uar_), polar bears (*N*_uma_), and divergence time between brown bear and polar bear populations (*T*). To encompass the genetic diversity of widely distributed brown bears, we considered 14 aDNA loci (18 brown bears and 18 polar bears) from Hailer et al. [3] and complete mtDNA sequences (9 brown bears and 26 polar bears) from Miller et al. [4] and GenBank (see Table S1). In this study, we used kernel-ABC to incorporate high dimensional summary statistics and improve approximation of posterior estimate given data [16], [17]. mtDNA genomes included multiple substitutions and did not follow the infinite site model in which each mutation occurs at a previously un-mutated site. We generated two mtDNA datasets by excluding sequences (Set-I) or multiple substitution sites (Set-II) and analyzed both datasets to evaluate the effects of sequence-editing methods on demographic inferences.

### Demographic History based on Autosomal DNA

To obtain posterior estimates of the demographic parameters given the aDNA data, we summarized the sequence data into SFS and HFS. Summary statistics were calculated for each locus and merged into a set of observed summary statistics across 14 loci (Figure S2). Based on observed data and prior conditions described above, we estimated posterior means of the parameters by kernel-ABC (Table 1). *N*_uar_ was estimated at 46,434 (standard deviation (S.D.), 520), which is approximately six times larger than *N*_uma_ = 7,867 (S.D. 252). Divergence time was estimated to be 136,597 (S.D. 2,264) generations ago, which was translated into 1.37 MYA based on an assumed generation time of 10 years. Nakagome et al. [5] showed that the time to most recent common ancestor in brown bears (*T*_MRCA-uar_) was more than *T* = 1.37 MYA in eight out of 13 loci, while *T*_MRCA_ in polar bears (*T*_MRCA-uma_) was less than the divergence time in most of these loci. Our population genetic analysis for aDNA genes supports the paraphyletic model of brown and polar bear lineages (Fig. 1).

**Table 1:** Posterior estimates of three demographic parameters.

Genes		Posterior means^a^		
		*N*_uar_	*N*_uma_	*T*
aDNA (14 loci)	Mean	46,434^b^	7,867^b^	136,597
	S.D.	520	252	2,264
mtDNA (Set-I)	Mean	67,132	2,351	30,992
	S.D.	1,405	113	607
mtDNA (Set-II)	Mean	57,777	4,232	32,847
	S.D.	726	41	337
*N*_mtDNA_/*N*_aDNA_	Set-I	0.723^c^	0.149^c^	
	Set-II	0.622^c^	0.269^c^	

a Distribution of posterior means for each parameter generated from 100 replications with 20,000 simulated samples.

b Effective population size based on aDNA is expressed as *N*_e_ individuals.

c Ratio of *N*_e_ based on mtDNA to aDNA was calculated by *N*_mtDNA_/2*N*_e._

**Figure 2:**
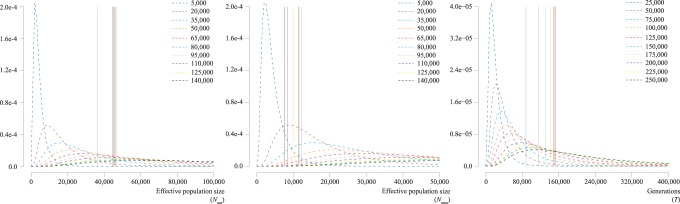
Plots of the posterior mean and prior density *N*_uar_, *N*_uma_, and *T* estimated by kernel-ABC. The horizontal axis indicates the effective population size or number of generations, while the vertical axis indicates the density of prior distributions. The prior density is shown as a dashed line, with mean (*μ*) and variance (*μ* ^2^). Ten different priors are distinguished by different colors and shown with *μ* values. Posterior means are shown as vertical solid lines, with colors corresponding to priors (see Table S2).

We examined the robustness of posterior estimates relative to prior distributions. We used a set of 10 different prior distributions for each demographic parameter and compared posterior means of the parameter among prior conditions (Fig. 2 and Table S2). For *N*_uar_, posterior estimates ranged from 44,790 (S.D. 1,076) to 46,633 (S.D. 707), except for a prior distribution with *LN*(*μ* = 5000, *μ* ^2^ = 5000^2^). In contrast, *N*_uma_ estimates ranged from 7,509 (S.D. 392) to 12,318 (S.D. 2,485). Divergence time ranged from 87,921 (S.D. 1,750) to 152,269 (S.D. 4,412). These results indicate that posterior estimates of the parameters are only weakly dependent on prior conditions, demonstrating the validity of Bayesian inference based on high-dimensional summary statistics.

### Demographic History based on mtDNA

To resolve gene tree discordance between aDNA and mtDNA, we estimated demographic parameters using mtDNA sequences. We used two mtDNA datasets (Set-I and Set-II) as the observed data in kernel-ABC. Set-I consists of 8,268 bp sequences from four brown bears and 10 polar bears, while Set-II includes 15,403 bp from nine brown bears and 26 polar bears (Table S1). Each of the datasets was summarized into a matrix of SFS (2D-SFS) (Figure S2). The estimated value of *N*_uar_/2 or *N*_uma_/2 was defined as the effective population size of mtDNA (*N*_mtDNA-uar_ or *N*_mtDNA-uma_). The posterior estimate of *N*_mtDNA-uar_ (Set-I: mean ± S.D. = 67,132±1,405; Set-II: 57,777±726) was approximately 29 or 14 times larger than *N*_mtDNA-uma_ (Set-I: 2,351±113; Set-II: 4,232±41) (Table 1). The divergence time between brown bear and polar bear populations (Set-I: 30,992±607 generations ago; Set-II: 32,847±337 generations ago) was estimated to be about 0.31 MYA and 0.33 MYA, respectively. These results suggest that posterior estimates from Set-I are compatible with those from Set-II and that our estimations do not likely depend on sequence-editing methods. Under the equilibrium assumptions of random mating, an equal number of males and females, and Poisson distribution of offspring, the effective population size based on mtDNA (*N*_mtDNA_) is expected to be a quarter of that based on aDNA (*N*_aDNA_). The ratio of *N*_mtDNA_ to *N*_aDNA_ (*N*_mtDNA_/*N*_aDNA_) in polar bears was 0.149 and 0.269 in Set-I and Set-II, respectively, which was relatively close to the expected value of 0.250 (Table 1). In contrast, the *N*_mtDNA_/*N*_aDNA_ ratio in brown bears was 0.723 and 0.622 in Set-I and Set-II, respectively, which was much larger than the expected value.

### Effects of Sex-biased Migration

Deviation from the expected ratio could have resulted from the following three forces: (i) differences in mutation rates between aDNA and mtDNA; (ii) natural selection on mtDNA; and (iii) sex-biased demographic events leading to different effective population sizes of males and females. For (i), we calculated mutation rates for aDNA and mtDNA based on the average number of substitutions between the giant panda and brown/polar bears. The number of substitutions in mtDNA was corrected using the Tajima-Nei distance model [24]. As expected, the mutation rate was higher in mtDNA (Set-I: 7.036×10^−8^ bp/site/generation; Set-II: 7.838×10^−8^ bp/site/generation) than in aDNA (1.314×10^−8^), although this difference was already incorporated into estimation of *N*_mtDNA_ and *N*_aDNA_. For (ii), we tested natural selection of mtDNA in brown bears using neutrality statistics (Table S3). However, none of the statistics showed signals indicating natural selection. Evolutionary forces of (i) and (ii) are unlikely to result in a high *N*_mtDNA-uar_/*N*_aDNA-uar_ ratio. For (iii), male-biased dispersal and female philopatry are known behavioral traits in brown bears [8], [9]. A recent study proposed the presence of sex-biased migration in ancestral populations of bears, which led to high or low genetic divergence of mtDNA or Y-linked DNA in bear speciation [10]. Female philopatry has been shown to increase *N*_mtDNA_ relative to *N*_aDNA_ [27], and male-biased migration has contributed to higher genetic differentiation in mtDNA than aDNA [28]. Hence, the observed *N*_mtDNA-uar_/*N*_aDNA-uar_ value can be explained by sex-biased migration in brown bears.

Female philopatry may lead to matriarchal population stratification in geographically different regions (Kenai, Kodiak, France, and ABC Islands). Based on Set-I and Set-II datasets, we considered mtDNA genealogy between each of the geographic lineages in brown bears and polar bears. First, we counted the number of mutations specific to brown bear, ancestral polar bear, or polar bear lineages (Figures S3 and S4). More mutations in the brown bear lineage have accumulated in Kenai or Kodiak, followed by France and the ABC Islands. Next, we estimated *T*_MRCA_ for each genealogy by kernel-ABC based on 2D-SFS. The *T*_MRCA_ estimate based on Set-I was much older among the Kenai and polar bear lineages (*T*_MRCA Kenai-uma_ = 1,119,160 years ago (ya), S.D. 8,860), followed by the France and polar bear lineages (*T*_MRCA France-uma_ = 688,207 ya, S.D. 8,018), and the ABC Islands and polar bear lineages (*T*_MRCA ABC-uma_ = 332,808 ya, S.D. 7,678). Similarly, we computed *T*_MRCA_ among polar bear lineages using SFS. The *T*_MRCA_ was 48,787 ya (S.D. 1,576), which was lower than estimates from brown/polar bear comparisons. All of the *T*_MRCA_ estimates based on Set-II were generally consistent with those based on Set-I (Figure S4). Therefore, the *T*_MRCA Kenai-uma_, *T*_MRCA France-uma_, and *T*_MRCA ABC-uma_ estimates likely reflect split times in the mtDNA genealogy between each of geographic lineages in brown bears and the ancestral lineage of polar bears. These results represent the highly differentiated mtDNA lineages of brown bears in each geographic region.

### Evolutionary History of Brown Bears and Polar Bears

**Figure 3:**
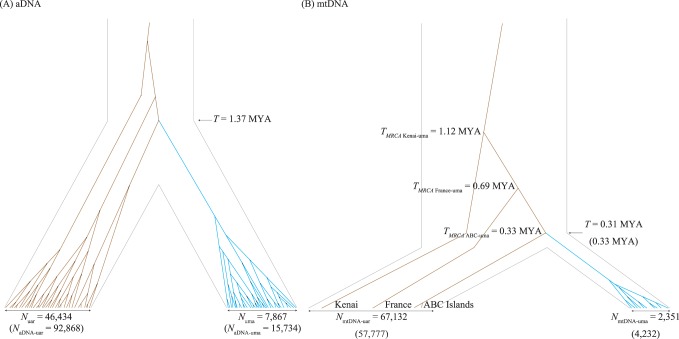
Demographic histories of brown and polar bears based on (A) aDNA and (B) mtDNA. (A) Parameters were estimated by kernel-ABC with the sequence data from brown bears (2*n* = 36) and polar bears (2*n* = 36) for 14 loci. The effective population size based on aDNA (*N*_aDNA-uar_ = 92,868 or *N*_aDNA-uma_ = 15,734) is twice the size of *N*_uar_ = 46,434 or *N*_uma_ = 7,867. Similarly, (B) demographic parameters were estimated from the Set-I dataset (8,268 bp from four brown bears and 10 polar bears). Parameters based on the Set-II dataset (15,403 bp from nine brown bears and 26 polar bears) are shown in parentheses. mtDNA genealogy is based on the results from the Set-I dataset. *T*_MRCA_ estimates are plotted on each MRCA between each of the geographic lineages in brown bears and the ancestral polar bear lineage (see Figure S3).

Biparentally inherited aDNA and matrilineal mtDNA show that the polar bear lineage derived from diverse brown bear lineages (Fig. 3). However, the divergence times between brown bear and polar bear populations are different between aDNA (1.37 MYA) and mtDNA (Set-I: 0.31 MYA; Set-II: 0.33 MYA) demographic histories. This discrepancy likely reflects different effects of sex-biased migration on aDNA and mtDNA genealogies. The aDNA genealogies are products of female philopatry and male-biased migration, while the mtDNA genealogy has only been shaped by female philopatry, and evolutionary traces of brown bears and polar bears have been recorded in mtDNA lineages. Geographic differentiations of brown bear lineages were shown in the mtDNA genealogy, although these patterns were likely homogenized in aDNA genealogies by male-biased migration between geographic regions, as suggested in a previous study [10]. Therefore, the mtDNA genealogy represents patterns of population divergence in the speciation history of polar bears from brown bears. The divergence time based on aDNA is close to *T*_MRCA Kenai-uma_ (Set-I: 1.12 MYA; Set-II: 1.20 MYA), showing that the ancestral lineage of polar bears in mtDNA genealogy split from the most distant brown bear lineage at this time. In contrast, the divergence time from mtDNA is almost equal to *T*_MRCA ABC-uma_ (Set-I: 0.33 MYA; Set-II: 0.42 MYA), suggesting that the ancestral lineage of polar bears split from the closest brown bear lineage at this time. A plausible explanation for the evolution of brown bears and polar bears is as follows: the initial divergence of brown bear lineages occurred at 1.12 MYA; geographic lineages of brown bears gradually diverged from the ancestral lineage of other brown bears and polar bears and expanded into broad geographic regions; after that, the polar bear lineage appeared from the ancestral lineage at 0.33 MYA and differentiated into the arctic region. This recent divergence is compatible with the oldest fossil record of polar bears, which is estimated to be between 110 ka and 130 ka [29]. Hence, the genetic diversity of brown bears and polar bears can be shaped by ancestral polymorphisms and sex-biased migration.

In conclusion, Bayesian inference based on the coalescent model highlights the fact that population demography underlies patterns of genetic variation across independent loci even though stochastic effects shape a unique genealogy at each locus. Furthermore, sex-biased migration has a significant effect on population demography [30], [31], and we observed that female philopatry and male-biased dispersal generated discordant genealogy in brown and polar bears between aDNA and mtDNA. aDNA, mtDNA, and sex-linked DNA have different modes of inheritance and thus reflect their own history of evolutionary demographics and natural selection. As population-based genomic sequencing becomes available to analyze a variety of wild animals, population genetic approaches can be applied to phylogenetic problems to model stochastic processes and inheritance characteristics involved in speciation history.

## Supporting Information

Figure S1 **Gene trees for 13 loci using aDNA sequence data.** Tree heights are proportional to *T*_MRCA_ scaled to millions of years ago (MYA). Black circles indicate mutations, and the numbers on the black circles correspond to the site No. in the total segregating sites. The number of haplotypes observed in brown bears (upper) and polar bears (lower) is shown at the base of the trees.
(EPS)

Figure S2
**Summary statistics for (a)–(n) aDNA (14 loci) and (o)–(p) mtDNA (Set I and Set II).** For (a)–(n), sequence data from aDNA genes are summarized as SFS_uar_ (8 bins), SFS_uma_ (8 bins), and 2D-HFS (48 bins). The number of bins is defined by Sturges’ formula 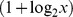, where *x* is the number of derived alleles or haplotypes. For SFS, the total number of alleles across 14 loci is 96 in brown bears with 7.6≈8 bins. For HFS, the number of haplotypes is 77 with 7.3≈7 bins. 2D-HFS is set as 7×7 bins, excluding one bin with a frequency of “0” (black). Derived allele frequencies are binned with 1–4, 5–8, 9–12, 13–16, 17–20, 21–24, 25–28, 29–36, while haplotype frequencies are binned with 0, 1–6, 7–12, 13–18, 19–24, 25–30, 31–36. For (o)–(p), mtDNA sequence data from Set I or Set II are summarized into 2D-SFS (38 bins or 89 bins). The number of bins in Set I or Set II is defined by the full spectra in brown bears (*n* = 4) and polar bears (*n* = 10) or by the full spectrum in brown bears (*n* = 9) and the given intervals in polar bears (0, 1–3, 4–6, 7–9, 10–12, 13–15, 16–18, 19–21, 22–24, 25–26). The bins with a frequency of “0” (Set I or Set II) and a fixed frequency (Set I) were excluded (black).(EPS)

Figure S3 ***T*** **_MRCA_ estimation in the genealogy of each of the geographic lineages in brown bear (Kenai, France, or ABC Islands) and polar bear lineages by kernel-ABC using the Set-I mtDNA dataset.** The number of mutations in a branch was counted in the brown bear lineage (brown branch), the ancestral lineage of polar bears (blue branch), or polar bear lineages (blue triangle). mtDNA sequence data are summarized into 2D-SFS (20 bins). *T*_MRCA_ posterior estimates are 1,119,160 ya (S.D. 8,860) for Kenai and polar bears, 688,207 ya (8,018) for France and polar bears, 332,808 ya (7,678) for ABC Islands and polar bears, and 48,787 ya (1,576) for polar bears.
(EPS)

Figure S4 ***T*** **_MRCA_ estimation in the genealogy of each of the geographic lineages in brown bear (Kenai, Kodiak, France, or ABC Islands) and polar bear lineages by kernel-ABC using the Set-II mtDNA dataset.** The number of mutations in a branch was counted in the brown bear lineage (brown branch), the ancestral lineage of polar bears (blue branch), or polar bear lineages (blue triangle). mtDNA sequence data are summarized into 2D-SFS (19 bins or 59 bins). *T*_MRCA_ posterior estimates are 1,200,084 ya (S.D. 4,849) for Kenai and polar bears, 1,196,608 ya (4,873) for Kodiak and polar bears, 733,916 ya (3,737) for France and polar bears, 415,548 ya (5,591) for ABC Islands and polar bears, and 94,899 ya (2,794) for polar bears.
(EPS)

Table S1 **Sequence information for aDNA genes and mtDNA sequences used in this study.**
(DOCX)

Table S2 **Posterior estimates of demographic parameters under different prior conditions.**
(DOCX)

Table S3 **Neutrality tests for mtDNA sequences from brown bears.**
(DOCX)
