# Supplementary material for: Ancestral Polymorphisms and Sex-Biased Migration Shaped the Demographic History of Brown Bears and Polar Bears
Source: PLoS One. 2013 Nov 13;8(11):e78813. doi: 10.1371/journal.pone.0078813 (PMC3827271; doi:10.1371/journal.pone.0078813)
Supplement: Table S1 — Sequence information for aDNA genes and mtDNA sequences used in this study. (DOCX) [file pone.0078813.s005.docx]

| **Table S1.** Sequence information for aDNA genes and mtDNA sequences used in this study. | | | |
| --- | --- | --- | --- |
| Gene | Locus | Accession numbers | |
|  |  | Brown bears | Polar bears |
| aDNA^a^ | *AZIN* intron 6 | HE657876 - HE657911 | HE657912 - HE657947 |
|  | *LRGUK* intron 14 | HE658306 - HE658341 | HE658342 - HE658377 |
|  | *SPTBN1* intron 31 | HE658392 - HE658427 | HE658428 - HE658463 |
|  | *ABCA1* intron 49 | HE658478 - HE658513 | HE658514 - HE658549 |
|  | *CCDC90B* intron 4 | HE658564 - HE658599 | HE658600 - HE658635 |
|  | *GGA3* intron 3 | HE658650 - HE658685 | HE658686 - HE658721 |
|  | *SCN5A* intron 24 | HE658736 - HE658771 | HE658772 - HE658807 |
|  | *ATP12A* intron 12 | HE658822 - HE658857 | HE658858 - HE658893 |
|  | *PREX2* intron 29 | HE657790 - HE657825 | HE657826 - HE657861 |
|  | *TRAPPC10* intron 8 | HE657962 - HE657997 | HE657998 - HE658033 |
|  | *SPTA1* intron 51 | HE658048 - HE658083 | HE658084 - HE658119 |
|  | *IGSF22* intron 15 | HE658134 - HE658169 | HE658170 - HE658205 |
|  | *SEL1L3* intron 20 | HE658220 - HE658255 | HE658256 - HE658291 |
|  | *OSTA* intron 5 | HE658908 - HE658943 | HE658944 - HE658979 |
|  |  |  |  |
| mtDNA^b^ |  | NC003427;  GU573491 (Kodiak);  JX196367 (Kenai)*;  EU497665 (France)*;  GU573489 (Baranof)*;  JX196368 (Baranof)*;  GU573486 (Admiralty);  GU573487 (Admiralty);  JX196369 (Admiralty) | JX196370*; JX196371*; JX196372; JX196373; JX196374; GU573490; GU573485; NC003428; JX196375*; JX196376; JX196377; JX196378*; JX196379; JX196380*; JX196381; JX196382; JX196383; JX196384; JX196385*; JX196386*; JX196387; JX196388; JX196389*; JX196390; JX196391*; JX196392* |
| ^a^Orthologous sequences of the giant panda were obtained from the genome assembly of "ailMel1 (GCA_000004335.1)" in the Ensembl Genome Browser (http://asia.ensembl.org/index.html). | | | |
| ^b^Sequences from one giant panda (EF212882) and two American black bears (JX196366; NC003426) were downloaded from GenBank (http://www.ncbi.nlm.nih.gov/genbank/). | | | |
| *Asterisk shows the Set-I sequences used after excluding sequences with multiple substitutions; geographic labels for brown bears are from Supplementary table 3 in Miller et al. (2012). | | | |
