# Supplementary material for: Ancestral Polymorphisms and Sex-Biased Migration Shaped the Demographic History of Brown Bears and Polar Bears
Source: PLoS One. 2013 Nov 13;8(11):e78813. doi: 10.1371/journal.pone.0078813 (PMC3827271; doi:10.1371/journal.pone.0078813)
Supplement: Table S2 — Posterior estimates of demographic parameters under different prior conditions. (DOCX) [file pone.0078813.s006.docx]

| **Table S2.** Posterior estimates of demographic parameters under different prior conditions. | | | |
| --- | --- | --- | --- |
| Demographic parameters | Prior conditions (*μ*) | Posterior estimates^a^ | |
|  | *LN*(*μ*, *μ*^2^) | Mean | S.D. |
| *N*_uar_ | 5,000 | 36,531 | 2,330 |
|  | 20,000 | 45,924 | 1,081 |
|  | 35,000 | 46,633 | 707 |
|  | 50,000 | 46,398 | 453 |
|  | 65,000 | 45,933 | 795 |
|  | 80,000 | 45,781 | 766 |
|  | 95,000 | 45,405 | 624 |
|  | 110,000 | 45,360 | 729 |
|  | 125,000 | 45,023 | 1,001 |
|  | 140,000 | 44,790 | 1,076 |
|  |  |  |  |
| *N*_uma_ | 5,000 | 7,509 | 392 |
|  | 20,000 | 8,377 | 522 |
|  | 35,000 | 8,358 | 384 |
|  | 50,000 | 8,404 | 991 |
|  | 65,000 | 7,552 | 1,816 |
|  | 80,000 | 9,802 | 2,233 |
|  | 95,000 | 11,337 | 2,860 |
|  | 110,000 | 10,307 | 3,784 |
|  | 125,000 | 12,318 | 2,485 |
|  | 140,000 | 11,540 | 4,243 |
|  |  |  |  |
| *T* | 25,000 | 87,921 | 1,750 |
|  | 50,000 | 116,063 | 3,906 |
|  | 75,000 | 131,353 | 3,914 |
|  | 100,000 | 142,286 | 2,200 |
|  | 125,000 | 150,664 | 3,754 |
|  | 150,000 | 151,865 | 4,867 |
|  | 175,000 | 152,269 | 4,412 |
|  | 200,000 | 150,016 | 2,286 |
|  | 225,000 | 150,122 | 7,053 |
|  | 250,000 | 148,240 | 6,016 |
| ^a^Distribution of posterior means for each parameter was generated from 100 replications with 10,000 simulated samples. | | | |
