# Supplementary material for: Ancestral Polymorphisms and Sex-Biased Migration Shaped the Demographic History of Brown Bears and Polar Bears
Source: PLoS One. 2013 Nov 13;8(11):e78813. doi: 10.1371/journal.pone.0078813 (PMC3827271; doi:10.1371/journal.pone.0078813)
Supplement: Table S3 — Neutrality tests for mtDNA sequences from brown bears. (DOCX) [file pone.0078813.s007.docx]

| **Table S3.** Neutrality tests for mtDNA sequences from brown bears. | | | | | |
| --- | --- | --- | --- | --- | --- |
|  |  | Tajima's D | Fu and Li's D | Fu and Li's F | Fay and Wu's H |
| Set-I (8,268 bp; brown bears: 4) | | | | | |
| Observed value |  | -0.01421 | 0.48813 | 0.46056 | -19.66667 |
| Coalescent simulation | Mean | -0.02309 | -0.13971 | -0.14426 | -0.06310 |
|  | 95% confidence interval  (lower limit) | -0.87009 | -1.63960 | -1.72870 | -82.00000 |
|  | 95% confidence interval  (upper limit) | 2.00914 | 2.20063 | 2.64098 | 47.00000 |
| *P*-value^a^ |  | 0.66160 | 0.80670 | 0.79280 | 0.19830 |
| Set-II (15,403 bp; brown bears: 9) | | | | | |
| Observed value |  | 0.27360 | 0.57045 | 0.59436 | -68.08333 |
| Coalescent simulation | Mean | -0.06682 | -0.16230 | -0.15785 | 0.72327 |
|  | 95% confidence interval  (lower limit) | -1.66327 | -1.87936 | -2.09477 | -254.22222 |
|  | 95% confidence interval  (upper limit) | 1.58831 | 1.51428 | 1.77328 | 102.27778 |
| *P*-value^a^ |  | 0.65350 | 0.73890 | 0.70690 | 0.14470 |
| ^a^*P*-values were calculated by comparing the fraction of simulated statistics that were less than the observed value. | | | | | |
